# Effects of Heat Acclimation and Acclimatisation on Maximal Aerobic Capacity Compared to Exercise Alone in Both Thermoneutral and Hot Environments: A Meta-Analysis and Meta-Regression

**DOI:** 10.1007/s40279-021-01445-6

**Published:** 2021-04-03

**Authors:** Mark Waldron, Rebecca Fowler, Shane Heffernan, Jamie Tallent, Liam Kilduff, Owen Jeffries

**Affiliations:** 1 A-STEM Centre, College of Engineering, Swansea University, Swansea, UK; 2 Welsh Institute of Performance Science, Swansea University, Swansea, UK; 3 School of Science and Technology, University of New England, Armidale, NSW Australia; 4 Sport, Health and Applied Sciences, St Mary’s University, London, UK; 5 School of Biomedical, Nutritional and Sport Sciences, Newcastle University, Newcastle Upon Tyne, UK

## Abstract

**Background:**

Heat acclimation and acclimatisation (HA) is typically used to enhance tolerance to the heat, thereby improving performance. HA might also confer a positive adaptation to maximal oxygen consumption ($V{\text{O}}_{2\max }$), although this has been historically debated and requires clarification via meta-analysis.

**Objectives:**

(1) To meta-analyse all studies (with and without control groups) that have investigated the effect of HA on $V{\text{O}}_{2\max }$ adaptation in thermoneutral or hot environments; (2) Conduct meta-regressions to establish the moderating effect of selected variables on $V{\text{O}}_{2\max }$ adaptation following HA.

**Methods:**

A search was performed using various databases in May 2020. The studies were screened using search criteria for eligibility. Twenty-eight peer-reviewed articles were identified for inclusion across four separate meta-analyses: (1) Thermoneutral $V{\text{O}}_{2\max }$ within-participants (pre-to-post HA); (2) Hot $V{\text{O}}_{2\max }$ within-participants (pre-to-post HA); (3) Thermoneutral $V{\text{O}}_{2\max }$ measurement; HA vs*.* control groups; (4) Hot $V{\text{O}}_{2\max }$ measurement, HA vs*.* control groups. Meta-regressions were performed for each meta-analysis based on: isothermal vs*.* iso-intensity programmes, days of heat exposure, HA ambient temperature (°C), heat index, HA session duration (min), ambient thermal load (HA session x ambient temperature), mean mechanical intensity (W) and the post-HA testing period (days).

**Results:**

The meta-analysis of pre–post differences in thermoneutral $V{\text{O}}_{2\max }$ demonstrated *small-to-moderate* improvements in $V{\text{O}}_{2\max }$ (Hedges’ *g* = 0.42, 95% CI 0.24–0.59, *P* < 0.001), whereas *moderate* improvements were found for the equivalent analysis of hot $V{\text{O}}_{2\max }$ changes (Hedges’ *g* = 0.63, 95% CI 0.26–1.00, *P* < 0.001), which were positively moderated by the number of days post-testing (*P* = 0.033, *β* = 0.172). Meta-analysis of control vs*.* HA thermoneutral $V{\text{O}}_{2\max }$ demonstrated a *small* improvement in $V{\text{O}}_{2\max }$ in HA compared to control (Hedges’ *g* = 0.30, 95% CI 0.06–0.54, *P* = 0.014) and this effect was larger for the equivalent hot $V{\text{O}}_{2\max }$ analysis where a higher (*moderate-to-large*) improvement in $V{\text{O}}_{2\max }$ was found (Hedges’ *g* = 0.75, 95% CI 0.22–1.27, *P* = 0.005), with the number of HA days (*P* = 0.018; *β* = 0.291) and the ambient temperature during HA (*P* = 0.003; *β* = 0.650) positively moderating this effect.

**Conclusion:**

HA can enhance $V{\text{O}}_{2\max }$ adaptation in thermoneutral or hot environments, with or without control group consideration, by at least a *small* and up to a *moderate–large* amount, with the larger improvements occurring in the heat. Ambient heat, number of induction days and post-testing days can explain some of the changes in hot $V{\text{O}}_{2\max }$ adaptation.

## Key Points

**Table:** 

Four meta-analyses were conducted to evaluate the effects of heat acclimation or acclimatisation (HA) on maximal oxygen consumption ($\dot{V}{\text{O}}_{2\max }$) and factors that moderate these effects.
The collective conclusions of the meta-analyses were that HA can enhance $\dot{V}{\text{O}}_{2\max }$ adaptation in thermoneutral (cool) or hot environments by at least a *small* and up to a *moderate–large* amount, with the descriptively largest improvements occurring in the heat.
The positive effects of HA on $\dot{V}{\text{O}}_{2\max }$ are apparent with or without the inclusion of a control group, suggesting its capacity to augment the effect of endurance training. Factors such as the level of ambient heat, the number of induction days and the post-testing days can partially moderate the change in hot $\dot{V}{\text{O}}_{2\max }$ adaptation.

## Introduction

Maximal aerobic capacity ($\dot{V}{\text{O}}_{2\max }$) is a well-established marker of cardio-respiratory fitness and related to long-term health outcomes [1–3], predicting longevity in a dose–response-dependent manner, alongside numerous other phenotypic changes [4]. In addition to the ‘protective’ health effects ascribed to cardiorespiratory fitness [5], $\dot{V}{\text{O}}_{2\max }$ is also a primary determinant of endurance performance, explaining ~ 20–60% of the variation in performances of different mode and distance, which can be realised by athletes when combined with other sub-maximal endurance performance determinants [6–8]. Despite the suggested limited trainability of $\dot{V}{\text{O}}_{2\max }$ [9], a number of different training approaches have been adopted to support its adaptation, such as repeated high-intensity or continuous endurance exercise, which typically confers *moderate* effects [10]. To augment these responses, or prepare individuals for maximal endurance exercise in hot conditions, there has been substantial interest in manipulation of environmental stressors, on the basis that exposure to a combination of exercise and hot environmental temperatures will exaggerate the effector response and subsequent stimulus for endurance adaptation [11].

Heat acclimation or acclimatisation (HA) describes processes of serial exposure to artificial or outdoor heated environments, respectively, often conducted in combination with exercise [12]. Engaging in HA enhances the capacity to thermoregulate in the heat, thus improving heat tolerance via the enhancement of various thermoregulatory mechanisms [13, 14]. Whilst it is largely agreed that HA improves exercise capacity and $\dot{V}{\text{O}}_{2\max }$ in the heat, its transfer to thermoneutral environments has been debated [15]. Some studies demonstrate 4–13% changes [16–21] and others report no change or a reduction following a range of HA protocols [22–28]. It was reasoned recently that insufficient post-HA adaptation periods could explain these discrepancies [21], alongside other factors, such as inter-individual differences in adaptation capacity [29]. It is possible that the HA dose (i.e. thermal load or training load) also explains the magnitude of the observed $\dot{V}{\text{O}}_{2\max }$ adaptation [30]. Indeed, heat acclimation is most commonly conducted in combination with exercise in an ‘isothermic’ (fixed period of time at a fixed pre-determined core temperature) or ‘iso-intensity’ mode (fixed exercise intensity), which can vary in duration but typically ranges between 4 and 14 days [12]. Iso-intensity modes are preferred for acclimatisation, owing to the less controllable environment, and their time scale is often longer to account for natural variation in the environment; however, this is thought to result in adaptations that are more specific to competition, if planned correctly [12, 14]. The selected type of heat exposure will drastically alter the subsequent stimuli for adaptation [12, 14]; however, it is not known how the selected HA modality and loading characteristics affect the adaptation of $\dot{V}{\text{O}}_{2\max }$ in hot or thermoneutral environments. Lastly, many HA studies have been appropriately questioned [31] for the absence of control groups (i.e. participants receiving no heat exposure) in their research design. This increases the risk of biased outcome estimates ascribed to HA and $\dot{V}{\text{O}}_{2\max }$ adaptation and requires further investigation.

To date, there have been meta-analyses conducted to evaluate the efficacy of HA on acclimation status and a number of physiological and performance outcomes [30, 32, 33]; however, while these articles provide detailed insights into broader questions regarding HA, their analytical focus has not been $\dot{V}{\text{O}}_{2\max }$ adaptation. This means that a substantial number of papers have been overlooked, and the potential for disparate conclusions on this important measure of cardiorespiratory fitness is possible. Thus, there has been no comprehensive meta-analysis of all HA studies to have measured $\dot{V}{\text{O}}_{2\max }$ as an outcome variable. Furthermore, no study has investigated the collective moderating effect of the abovementioned factors, such as HA mode, thermal or training load and post-testing periods on $\dot{V}{\text{O}}_{2\max }$ adaptation. Owing to the historical debate of this topic and ongoing consistency of evidence, we sought to meta-analyse all studies (with and without control groups) that have investigated the effect of HA on $\dot{V}{\text{O}}_{2\max }$ adaptation in thermoneutral or hot environments. We also performed a number of meta-regressions to establish the moderating effect of selected variables on the variability in $\dot{V}{\text{O}}_{2\max }$ adaptation.

## Methods

### Search Strategy

All literatures that investigated the effect of HA on $\dot{V}{\text{O}}_{2\max }$ were searched and obtained using the Preferred Reporting Items for Systematic Reviews and Meta-Analyses (PRISMA) guidelines [34], with a pre-determined search strategy. Thus, a literature search was conducted using the key search terms “heat acclimation, $\dot{V}{\text{O}}_{2\max }$ and thermoneutral”. First-order search terms used were: “heat acclimation”, “acclimatisation”, “heat acclimatization” and “acclimatization”. Second-order search terms used were: “$\dot{V}{\text{O}}_{2\max }$”, “exercise performance”, “aerobic capacity”, “maximal oxygen uptake”, “maximal aerobic power” and “temperate exercise performance”. Searches were performed across four databases: PubMed, SPORTDiscus, SciDirect and Google Scholar. Other sources include social media and the reference lists of selected studies. Searches were conducted between the 6 and 8 May 2020. Following initial screening of databases, references of included studies were screened against inclusion and exclusion criteria by two authors (RF and MW) to obtain any additional studies missed from the database searches.

### Selection Criteria

**Fig. 1:**
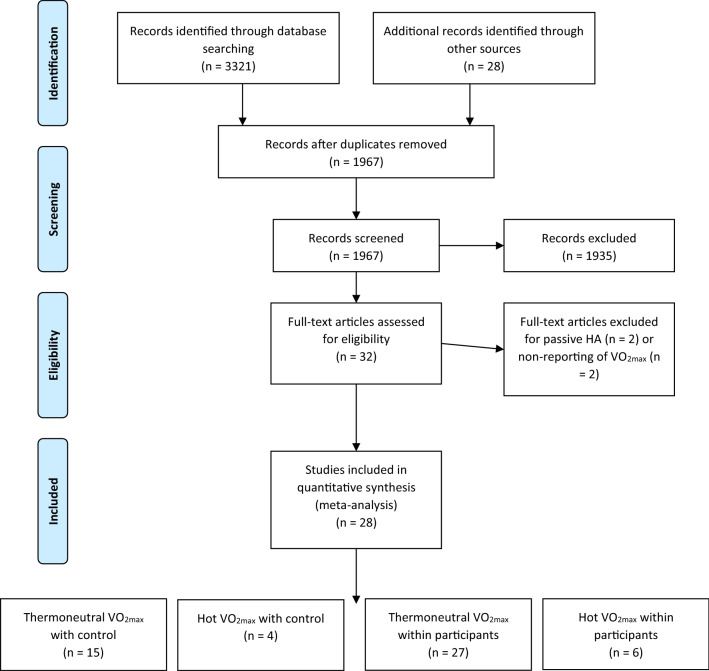
Overview of the systematic review and selection process. Some articles contributed to more than one meta-analysis owing to their designs. *V*O_2max_ maximal oxygen consumption, *HA* heat acclimation or acclimatisation

The full text of each paper was assessed separately, by two authors (RF and MW), against the below criteria to determine suitability for inclusion in the systematic review. Figure 1 illustrates the stages of selection criteria, according to the PRISMA guidelines for meta-analysis and systematic review [35]. Papers were only selected for quantitative synthesis if they satisfied the following inclusion criteria: (a) the full text was written in the English language and published in a peer-reviewed scientific journal; (b) human participants of any training background, health or gender; (c) active heat acclimation or acclimatisation of any form was utilised; (d) outcome measurements ($\dot{V}{\text{O}}_{2\max }$) were performed pre- and post-HA. Abstracts and conference proceedings, PhD dissertations, letters, and reviews were excluded.

### Classification of Studies

Following the exclusion of four studies, after assessment of full-text articles for eligibility, 28 peer-reviewed studies met the inclusion criteria and were categorised by the researchers into four groups, based on study design (Fig. 1), with four subsequent meta-analyses conducted, some of which necessarily included different aspects of the same study twice. The grouping of studies was as follows:

1. Thermoneutral $\dot{V}{\text{O}}_{2\max }$ within-participants (pre-to-post HA intervention).
2. Hot $\dot{V}{\text{O}}_{2\max }$ within-participants (pre-to-post HA intervention).
3. Thermoneutral $\dot{V}{\text{O}}_{2\max }$ measurement, with comparison between intervention and control groups.
4. Hot $\dot{V}{\text{O}}_{2\max }$ measurement, with comparison between intervention and control groups.

### Quality Assessment

**Fig. 2:**
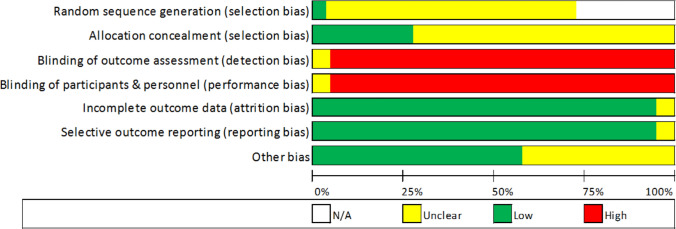
Risk of bias assessment

Quality assessment of all 28 included articles was completed independently by two authors (RF and MW) to assess for risk of bias. These were cross-checked for agreement. Seven assessment criteria were used to assess for risk of bias under the Cochrane Review criteria [36]: (1) sequence generation, (2) allocation concealment, (3) blinding of participants, (4) blinding of outcome data, (5) incomplete outcome data, (6) selective outcome reporting, and (7) other sources of bias. Each assessment criterion was judged by the review authors under three classifications, “Yes” to indicate low risk of bias, “No” to indicate high risk of bias and finally, “Unclear” to indicate level of bias is unclear or not known (Fig. 2).

### Data Extraction

Data were extracted independently by two authors (RF and MW) and entered into a custom Excel spreadsheet for cross-checking. Collected data included: (1) characteristics of the sample (sex, health status, training status); (2) study design [isothermal or iso-intensity]; (3) HA length [days]; (4) HA session duration [min]; (5) physiological intensity; (6) mechanical intensity [W, as stated or derived] (7) HA temperature [ambient °C]; (8) HA index; (9) core temperature [**°**C]; (10) ambient thermal load [ambient temperature × session duration]; (11) post-testing period [days] and (12) $\dot{V}{\text{O}}_{2\max }$ (mL/kg/min) (Tables 1 and 2). Further descriptions of selected or derived variables are included in later sections.

**Table 1:** Study characteristics for studies with thermoneutral maximal oxygen uptake ($\dot{V}{\text{O}}_{2\max }$) measurements following heat acclimation/acclimatisation (HA)

Study	Design and participants	HA length (days)	HA session duration (min)	Physiological intensity	Mech. intensity (W)	HA temp. (°C)	HA index	Core temp. (°C)	Ambient thermal load (AU)	Post- testing period (days)
Aoyagi et al. [37]	Acclimation of healthy male students & military personnel, ranging from sedentary to moderately active	6	60	50% $\dot{V}{\text{O}}_{2\max }$	140	40.0	43.0	–	2400	3^a^
Gore et al. [38]	Acclimatisation of trained male & female rowers	28	160	18–22 strokes/min	–	25.5	25.50	–	4080	7^a^
Heled et al. [39]	Acclimation of young, healthy males	12	120	30% $\dot{V}{\text{O}}_{2\max }$	109	40.0	48.0	–	4800	–
Karlsen et al. [24]	Acclimatisation of well-trained male cyclists	14	180	60–100% HR_max_ & ‘moderate’ intensity	–	34.1	32.0	39.0	6138	3.5
Mikkelsen et al. [31]	Acclimation of well-trained, sub-elite male cyclists	28^b^	60	60% $\dot{V}{\text{O}}_{2\max }$	204	37.5	38.5	38.5	2250	6^a^
Machado-Moreira et al. [40]	Acclimation of healthy physically active young males	9	60	50% $\dot{V}{\text{O}}_{2\max }$	150	40.0	44.0	–	2400	–
Nadel et al. [16]	Acclimation of relatively unfit males	10	60	75% $\dot{V}{\text{O}}_{2\max }$	180	45.0	39.0	–	2700	2^a^
Neal et al [26]	Acclimation of trained male endurance athletes	11	82	ISO	103	40.0	55.0	38.5	3280	2^a^
Neal et al. [41]	Acclimation of performance level 3 trained male cyclists and triathletes	5	90	ISO	102	40.0	55.0	38.6	3600	2^a^
Pivarnik et al. [19]	Acclimation of healthy, untrained males	6	90	60% $\dot{V}{\text{O}}_{2\max }$	138	40.0	46.0	–	3600	–
Rendell et al. [27]	Acclimation of performance level 3 trained male endurance athletes	11	82	ISO	109	40.0	55.0	38.5	3280	2^a^
Rivas et al. [42]	Acclimation of healthy untrained males & females	10	90	–	52	42.0	46.0	38.5	3780	1
Ronnestad et al. [43]	Acclimatisation of elite male cyclists	24^b^	60	~ 45% of 4 mmol B[La]	137	38.0	58.5	–	2280	–
Shvartz et al. [17]	Acclimation of healthy trained, young males	8	180	120–166 beats/min	41	39.4	52.8	37.7–37.9	7092	1
Takeno et al. [44]	Acclimation of healthy males	10	60	60	138	30.0	31.0	–	1800	2^a^
Tebeck et al. [45]	Acclimation of well-trained male cyclists	5	100	ISO	211	44.5	45.0	38.5	4450	3
Travers et al. [46]	Acclimation of trained male cyclists & triathletes	10	90	65	152	40.0	48.0	38.4	3600	10^a^
Van de Velde et al. [47]	Acclimation of males & female endurance-trained athletes	14	–	Unknown iso–intensity	–	–	–	–	–	2^a^
Van de Velde et al. [48]	Acclimation of males & female endurance-trained athletes	60	30	55–60% HRR	–	–	–	–	–	1
Waldron et al. [21]	Acclimation of young, healthy, trained amateur cyclists	10	60	50	189	38.0	39.0	38.5	2280	4
White et al. [49]	Acclimation of well-trained male cyclists	10	120	55	200	40.0	39.0	–	4800	8^c^
Willmott et al. [50]	Acclimation of moderately-trained performance level 3 males	10	60	ISO	128–131	45.0	47.0	38.2–38.3	2700	3

Some articles are included in more than one meta-analysis, depending on the study designs

*ISO* isothermal model, *HR* heart rate, *HRmax* maximal heart rate, *HRR* heart rate reserve, *B[La]*  blood lactate concentration, *Temp.*  temperature, *AU* arbitrary unit, *Mech.*  mechanical

^a^Maximum period

^b^Intermittent HA across the stated time period

^c^Average period

**Table 2:** Study characteristics for studies with hot maximal oxygen uptake ($\dot{V}{\text{O}}_{2\max }$) measurements following heat acclimation/acclimatisation (HA)

Study	Design and participants	HA length (days)	HA session duration (min)	Physiological exercise intensity	Mech. intensity (W)	HA temp. (°C)	HA index	Core temp. (°C)	Ambient thermal load (AU)	Post-testing period (days)
Chen et al. [23]	Acclimation of male students from the national all-star table tennis and badminton team	5	35	10% < GET to 10% > GET	–	38.4	51.0	–	1344	1
James et al. [51]	Acclimation of amateur male & female runners	5	90	ISO	201	36.6	48.0	38.5	3294	7^a^
Keiser et al. [25]	Acclimatisation of well-trained males	10	90	50%$\dot{V}{\text{O}}_{2\max }$	170	38.0	39.0	–	3420	5^a^
Lorenzo et al. [20]	Acclimation of highly trained male & female endurance cyclists	10	45	50%$\dot{V}{\text{O}}_{2\max }$	184.5	40.0	43.0	–	1800	7^a^
Sawka et al. [18]	Acclimation of male soldiers	9	120	40–50% $\dot{V}{\text{O}}_{2\max }$_x_	133	49.0	55.0	–	5880	1
Sotiridis et al. [52]	Acclimation of healthy, moderately active-to-trained males	10	90	ISO	115	35.0	41.0	38.5	3150	3^a^

Some articles are included in more than one meta-analysis, depending on the study designs

*GET* gas exchange threshold, *ISO* isothermal model, *Temp.*  temperature, *AU* arbitrary unit, *Mech.*  mechanical

^a^Maximum period

For the two within-group (no control group) meta-analyses, the pre- and post-HA mean ± SD $\dot{V}{\text{O}}_{2\max }$ values were extracted and standardized mean differences (SMD) based thereon. For the two between-group (experimental vs. control group) meta-analyses, the post-HA or control training mean ± SD $\dot{V}{\text{O}}_{2\max }$ values were extracted and SMD based thereon. The analysis of post-test scores was preferred over ‘change scores’ owing to the inconsistency of reporting across selected studies and the resulting over-reliance on SD imputing. However, baseline differences in $\dot{V}{\text{O}}_{2\max }$ were not reported across the selected studies in the control group meta-analyses conducted herein. Furthermore, adopting meta-analysis of change scores has also been questioned for reasons relating to outcome sensitivity and exaggeration of between-subject variances [53, 54]. Publicly available software (WebPlotDigitizer, Version 3.12) was used to extrapolate any unreported values from figures to raw mean and SD data. Both crossover and independent groups designed were considered for the control group meta-analyses. In three instances, where the experimental group was separated into independent groups within the same study, all comparisons were used in the meta-analysis [17, 38, 50].

For consistency across all of our analyses, where $\dot{V}{\text{O}}_{2\max }$ was reported in absolute terms (i.e. mL/min or L/min), the closest reported mean body mass (kg) to the $\dot{V}{\text{O}}_{2\max }$ measurement was used to determine the relative $\dot{V}{\text{O}}_{2\max }$ (mL/kg/min) and the SD was proportionally inferred. Across all studies included, direct assessments of $\dot{V}{\text{O}}_{2\max }$ were performed using open-circuit spirometry in a laboratory. In one instance where insufficient SD data of pre- and post-test $\dot{V}{\text{O}}_{2\max }$ were reported [51], the baseline (pre) SD was carried over. This was deemed to be more accurate than imputing data based on dispersions from the other studies in the meta-analysis [36] and avoided the exaggeration of the SD, which would influence the SMD result in the meta-analysis. Instances where papers had more than two experimental groups, due to additional interventions, they were matched accordingly to control or within-participant analysis. Where groups used permissive dehydration, only the data from the euhydrated participants were used in the within-participants analysis.

### Statistical Analysis

The meta-analysis was performed in RStudio (version 4.0.0, 2020, RStudio, Inc. software, Boston, MA), using the ‘metafor’ package. Fixed-effects models were preferred for all analyses, owing to the homogenous nature of the samples across selected studies [55]. Descriptive data are reported as mean ± standard deviation (SD) throughout. All effects are reported using Hedges’ g standardized mean differences (SMD ± 95% confidence intervals; CIs) across the four different meta-analyses. Heterogeneity was assessed using the *I*^2^ statistic.

Moderator analyses (meta-regressions) were conducted to explain additional variability in the SMD of $\dot{V}{\text{O}}_{2\max }$_._ We used weighted, random-effect meta-regressions, with maximum likelihood estimation [56]. The candidate moderators were selected based on their theorised contribution to the acclimated or endurance-adapted phenotype [11, 12]. In some cases, not all moderators could be used, owing to insufficient (< 50% data) or inconsistent data reporting. Statistical interactions between moderators were not considered for the analyses. The moderators considered for inclusion in the meta-analyses were: acclimation/acclimatisation model (isothermal vs*.* iso-intensity), HA days (total days of heat exposure), HA ambient temperature (°C), heat index [57], HA session duration (time [min] of each HA session per visit), mean core body temperature (°C), ambient thermal load (HA session x ambient temperature), mean mechanical intensity of HA exercise (W) and the post-HA testing period (days). For isothermal protocols, the session duration was calculated based on the entire session, rather than when the target core temperature was reached. Post-testing days refer to the number of days after the HA intervention that $\dot{V}{\text{O}}_{2\max }$ testing took place and were recorded as reported in the manuscript as either the exact number or the maximum number of days reported. While efforts were made to describe every study consistently, some approximations were necessary for mechanical intensity. After data extraction, mean core body temperature was removed owing to consistently insufficient data. We considered it necessary to have some common indication of exercise intensity, given its potential importance for endurance adaptation [58]. For example, where mechanical power output was unreported during HA, it was linearly extrapolated from the reported fraction of $\dot{V}{\text{O}}_{2\max }$ maintained during HA and corresponding peak power output values from the a priori graded exercise test [20, 25, 40, 41]. In the uncommon cases where the mechanical power output was not available during the HA or in the graded exercise test, the reported metabolic power was used to derive mechanical power using common gross efficiency values [16, 37–39, 49] for that exercise mode (inclined walking ~ 30% [59]; cycling ~ 20%; [60]). Finally, where mixed exercise designs were adopted in studies, a weighted mean value of mechanical power output was calculated, based on the time spent in different exercise modes [26, 27, 42].

Publication bias plots (i.e. funnel plots) were produced in RStudio (version 3.5.2, 2020, RStudio, Inc. software, Boston, MA), where the relationship between the effect size and the standard error of each data-set was visually inspected. Thereafter, Egger’s test was conducted on all meta-analytic data-sets, with significant (*P* < 0.05) results leading to Duval and Tweedie’s trim and fill correction [61]. The adjusted meta-analysis and funnel plot were then used for the current analysis. Across all analyses, the magnitudes of the effects were assessed based on the thresholds of: 0.2, 0.5 and 0.8 for *small, moderate and large*, respectively [62]. The effect of moderator variables was assessed based on the magnitude of the slope (*β*). In all statistical analyses, a value of *P* < 0.05 was considered as a significant difference.

## Results

### Within-Subject Pre–post Differences in Thermoneutral $\dot{V}{\text{O}}_{2\max }$

**Fig. 3:**
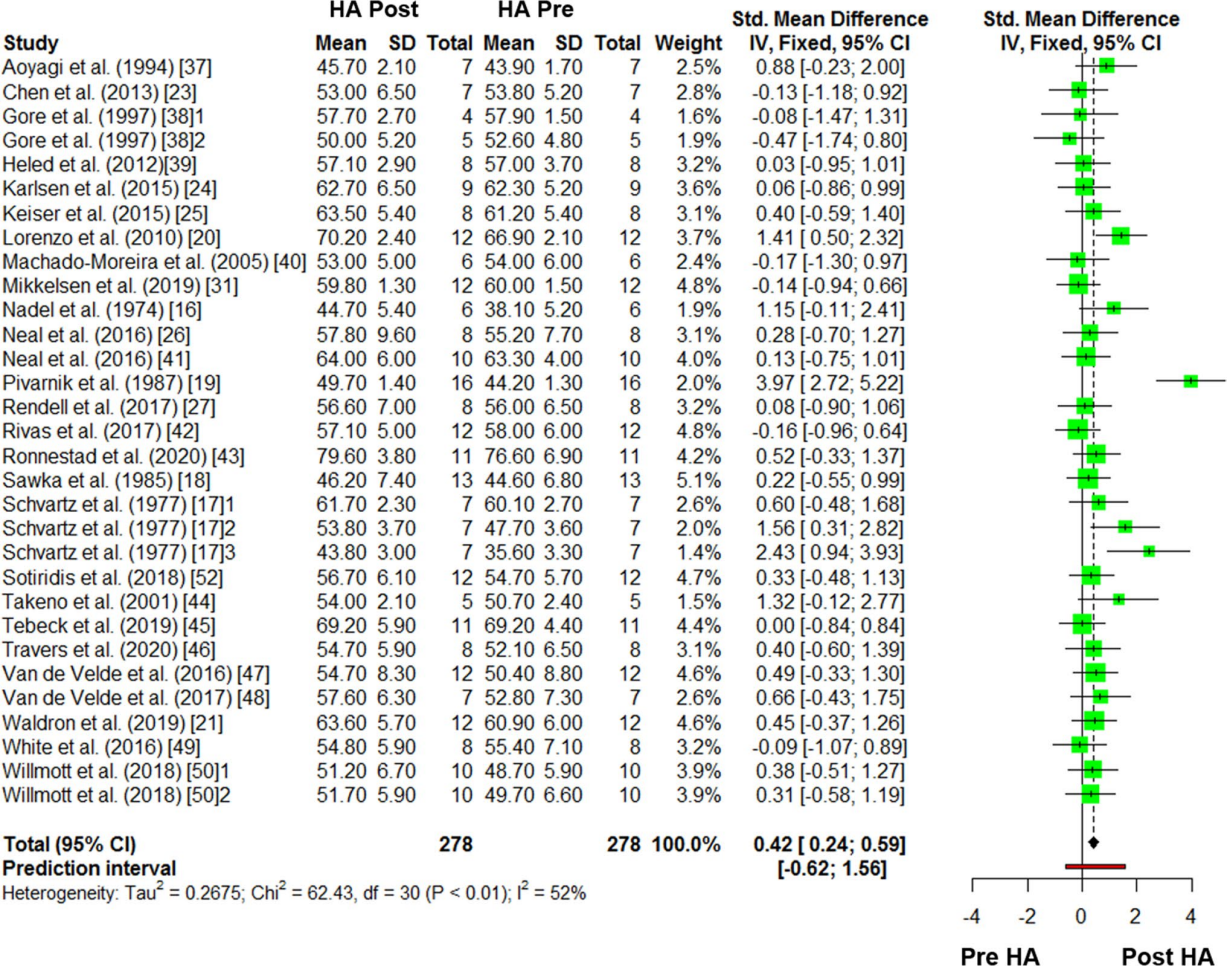
The pre-to-post, within-subject changes in thermoneutral maximal oxygen consumption ($\dot{V}{\text{O}}_{2\max }$) following heat acclimation (HA). *SMD* standardised mean differences, total = sample size (see throughout). Numbers following the referenced article denote a separate comparison within the same study

The results for the meta-analysis of pre–post differences in thermoneutral $\dot{V}{\text{O}}_{2\max }$ are reported in Fig. 3. There was a *small-to-moderate* improvement in $\dot{V}{\text{O}}_{2\max }$ from pre-to-post HA (Hedges’ g = 0.42, 95% CI 0.24–0.59, *P* < 0.001). The *I*^2^ statistic demonstrated 51.9% heterogeneity. There was no effect (*P* > 0.05) of any moderator variables on this meta-analysis (Table 3).

**Table 3:** Candidate moderators of maximal oxygen consumption changes following heat acclimation or acclimatisation (HA) across four meta-analyses

Variable	Within thermoneutral		Within hot		Control thermoneutral		Control hot	
	Slope (*β*) (± 95% CI)	*P*-value	Slope (*β*) (± 95% CI)	*P*-value	Slope (*β*) (± 95% CI)	*P*-value	Slope (*β*) (± 95% CI)	*P*-value
HA model type	0.340 (– 0.251 to 0.933)	0.259	0.385 (– 0.785 to 1.556)	0.519	– 0.015 (– 1.105 to 1.074)	0.978	1.384 (– 0.435 to 3.204)	0.135
HA days	– 0.027 (– 0.006 to 0.009)	0.134	0.111 (– 0.131 to 0.355)	0.372	– 0.016 (– 0.071 to 0.038)	0.569	0.290 (0.049 to 0.532)	0.018*
HA ambient temperature	0.015 (– 0.043 to 0.074)	0.608	– 0.010 (– 0.134 to 0.114)	0.870	0.052 (– 0.019 to 0.121)	0.152	0.650 (0.211 to 1.089)	0.003*
Heat index	0.040 (– 0.011 to 0.082)	0.113	– 0.050 (– 0.140 to 0.040)	0.279	0.030 (– 0.010 to 0.071)	0.145	– 0.110 (– 0.319 to 0.094)	0.290
HA session duration	0.000 (– 0.005 to 0.006)	0.846	– 0.008 (– 0.026 to 0.009)	0.367	– 0.002 (– 0.009 to 0.005)	0.644	– 0.191 (– 0.064 to 0.026)	0.407
Ambient thermal load	0.000 (– 0.000 to 0.000)	0.592	– 0.000 (– 0.005 to 0.002)	0.421	– 0.000 (– 0.002 to 0.000)	0.987	– 0.000 (– 0.002 to 0.000)	0.472
Mean mechanical intensity of HA	– 0.003 (– 0.009 to 0.004)	0.401	0.011 (– 0.006 to 0.027)	0.199	– 0.000 (– 0.008 to 0.008)	0.984	0.001 (– 0.006 to 0.006)	0.942
Post-HA testing period	– 0.025 (– 0.107 to 0.057)	0.546	0.172 (0.013 to 0.330)	0.033*	0.033 (– 0.159 to 0.225)	0.737	0.079 (– 0.394 to 0.554)	0.742

*Significant (*P* < 0.05) moderating effect

### Within-Subject Pre–post Differences in Hot $\dot{V}{\text{O}}_{2\max }$

**Fig. 4:**
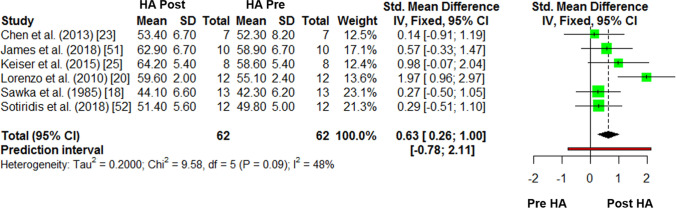
The pre-to-post, within-subject changes in hot maximal oxygen consumption ($\dot{V}{\text{O}}_{2\max }$) following heat acclimation (HA)

The results for the meta-analysis of pre–post differences in hot $\dot{V}{\text{O}}_{2\max }$ are reported in Fig. 4. There was a *moderate* improvement in $\dot{V}{\text{O}}_{2\max }$ from pre-to-post HA (Hedges’ *g* = 0.63, 95% CI 0.26–1.00, *P* < 0.001). The *I*^2^ statistic was 47.8%. The number of days post-testing was a significant moderator of the SMD in $\dot{V}{\text{O}}_{2\max }$ (*P* = 0.033, *β* = 0.172), indicating that for every day after HA, a ~ 0.17 standardised increase in $\dot{V}{\text{O}}_{2\max }$ is estimated (range 1–7 days). There was no effect (*P* > 0.05) of any other moderator variables on this meta-analysis (Table 3).

### Control vs. HA Differences in Thermoneutral $\dot{V}{\text{O}}_{2\max }$

**Fig. 5:**
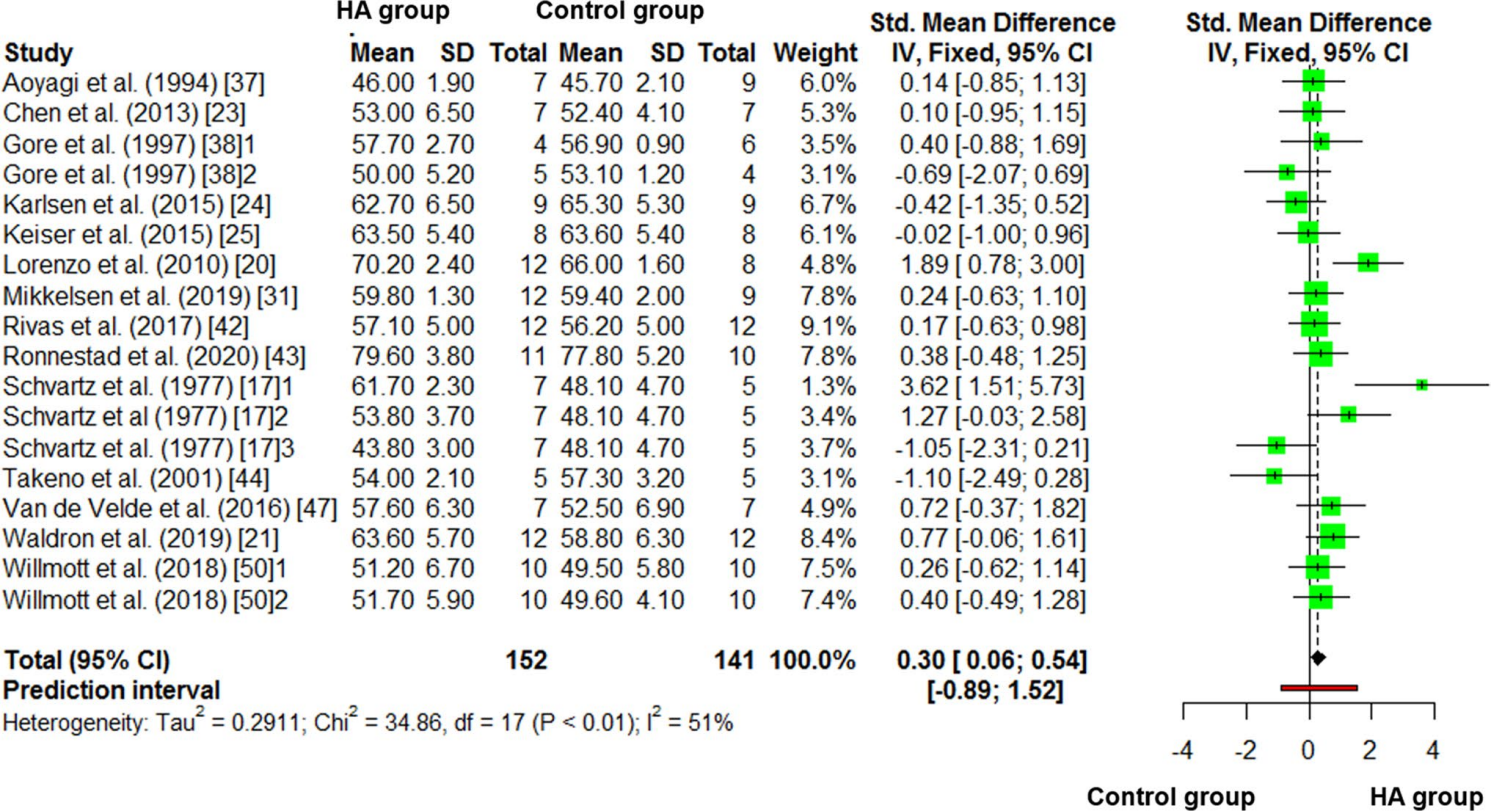
Control vs*.* heat acclimation (HA) post-intervention comparison of thermoneutral maximal oxygen consumption ($\dot{V}{\text{O}}_{2\max }$). Numbers following the referenced article denote a separate comparison within the same study

The results for the meta-analysis of control vs. HA thermoneutral $\dot{V}{\text{O}}_{2\max }$ are reported in Fig. 5. There was a higher (*small*) improvement in $\dot{V}{\text{O}}_{2\max }$ in HA compared to control (Hedges’ *g* = 0.30, 95% CI 0.06–0.54, *P* = 0.014). The *I*^2^ statistic was 51.2%. There was no effect (*P* > 0.05) of any moderator variables on this meta-analysis (Table 3).

### Control vs. HA Differences in Hot $\dot{V}{\text{O}}_{2\max }$

**Fig. 6:**
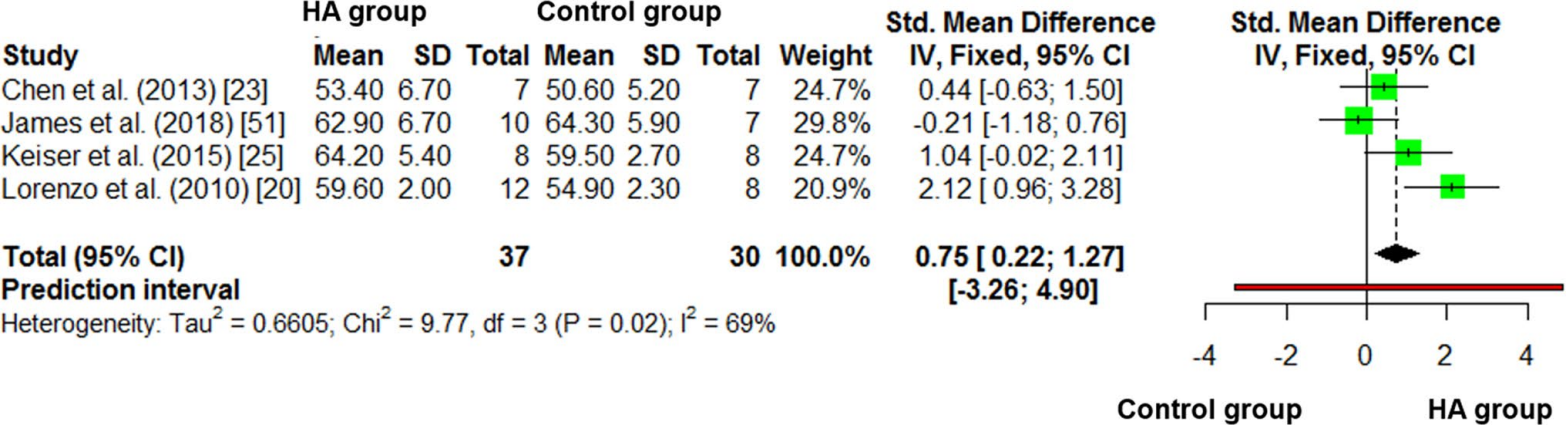
Control vs*.* heat acclimation (HA) post-intervention comparison of hot maximal oxygen consumption ($\dot{V}{\text{O}}_{2\max }$)

In the control vs. HA meta-analysis of hot $\dot{V}{\text{O}}_{2\max }$ (Fig. 6), there was a higher (*moderate-to-large*) improvement in $\dot{V}{\text{O}}_{2\max }$ in HA compared to control (Hedges’ *g* = 0.75, 95% CI 0.22–1.27, *P* = 0.005). The *I*^2^ statistic was 69.3%. Both the number of HA days (*P* = 0.018; *β* = 0.291) and the ambient temperature during HA (*P* = 0.003; *β* = 0.650) were moderators of this effect (Table 3), indicating a greater change in $\dot{V}{\text{O}}_{2\max }$ in hotter ambient temperatures and longer acclimation periods.

### Risk of Bias

**Fig. 7:**
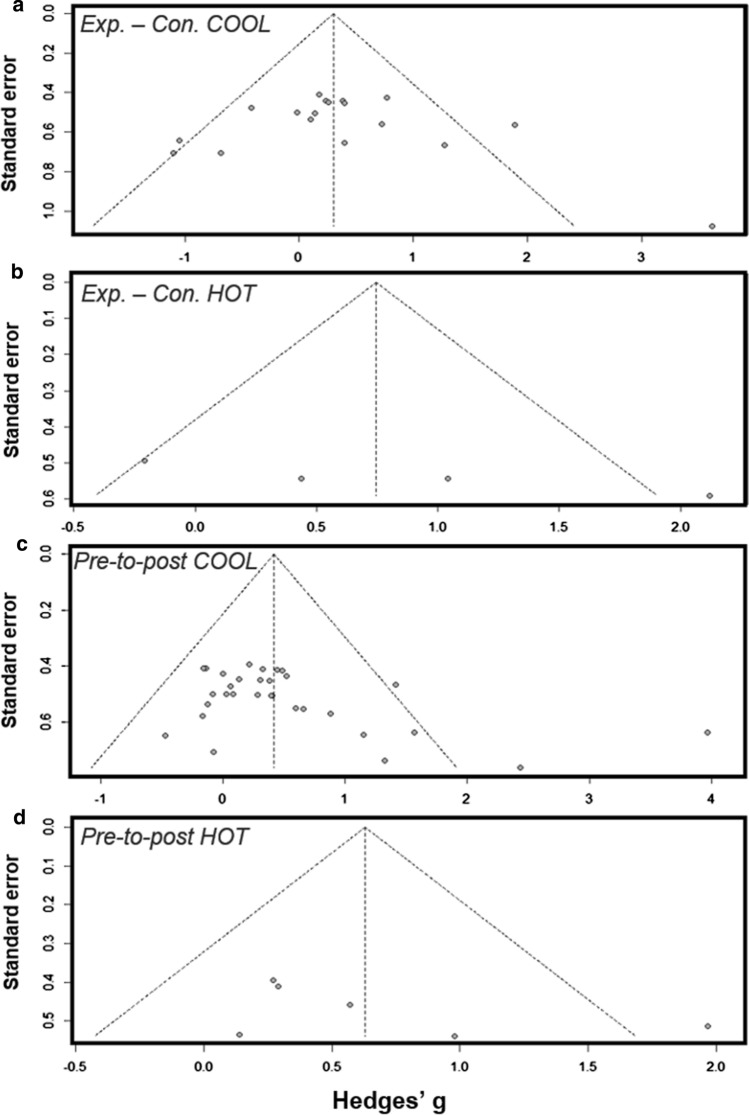
Risk of bias plots for measures of maximal oxygen consumption. **a** Heat acclimation (HA) vs*.* control in thermoneutral environments, **b** HA vs. control in hot environments, **c** pre-to-post changes in thermoneutral environments and **d** pre-to-post changes in hot environments. Hedges’ *g* = the standardised mean difference. *Exp.*  experimental, *Con* control

The studies included had a generally low risk of bias, with outcome data reported thoroughly and completely for the purposes of the current meta-analyses. Given the nature of HA, there were necessary risks of blinding and in selected studies, only experimental groups were chosen, thus voiding random sequencing (Fig. 2). Figure 7 shows that publication bias analysis (standard mean differences and standard error relationship) was generally symmetrical, with minimal outliers [see sect. 2.6 for trim and fill procedures, which were applied to panels a and c (Fig. 7)].

## Discussion

We conducted four meta-analyses to evaluate the efficacy of HA on $\dot{V}{\text{O}}_{2\max }$ adaptation in thermoneutral or hot environments, as well as establishing the moderating effect of selected variables on the magnitude of adaptation reported across studies. This has particular relevance for those intending to utilise HA to increase aerobic capacity in cool or hot environments, such as athletes or military personnel. Across all meta-analyses, there was an improvement in $\dot{V}{\text{O}}_{2\max }$ following HA, ranging from 0.30 (*small*) to 0.76 (*moderate-large*) standardised mean changes. These significant improvements were found, irrespective of the hot or thermoneutral environment used during testing, although there were stronger effects in the hot $\dot{V}{\text{O}}_{2\max }$ test results. This is consistent with the general recommendation that hot training confers adaptations to performance tests in environments that mimic that of training [63] and were anticipated based on the principle of environmental training specificity [64, 65]. Indeed, cardiovascular adaptations to HA (see [66]) should more thoroughly prepare participants for the severe blood flow conflicts experienced during hot exercise, as compared to thermoneutral [67], and could explain the descriptively larger trend in hot $\dot{V}{\text{O}}_{2\max }$ changes in the heat across the meta-analyses conducted here. This is consistent with the understanding that $\dot{V}{\text{O}}_{2\max }$ is primarily limited by central factors (i.e. cardiac output and skeletal muscle blood flow [68])—reductions of which also impair performance in the heat [67]. However, the finding of greatest importance revealed herein is that cross-adaptation does occur, with training in the heat augmenting the effect of exercise when targeting thermoneutral $\dot{V}{\text{O}}_{2\max }$ changes. This finding has implications for cardiorespiratory health in the general population [4] and contributes methods with which to enhance the classically described model of endurance performance determinants [69].

The consistent, positive effect of HA on $\dot{V}{\text{O}}_{2\max }$ across the within- and between-participant meta-analyses implies that the absence of a control group (those not receiving HA) does not appear to substantially affect the outcome. Comparison of between- (controlled) and within-participant meta-analyses analyses demonstrated that both the thermoneutral (Hedges’ *g* = 0.42 and 0.30, respectively) and hot (Hedges’ *g* = 0.63 and 0.75, respectively) resulted in similar changes in $\dot{V}{\text{O}}_{2\max }$. These results should not encourage the use of within-participant time-series designs but should arrest the debate [15] that HA is equivalent to thermoneutral training in eliciting $\dot{V}{\text{O}}_{2\max }$ adaptations, when all studies are considered.

On the basis that the mode of HA could elicit different physiological responses, we conducted sub-analyses to determine the potential moderating effect of isothermal or iso-intensity programmes, which are most commonly adopted in the available literature. However, there was no effect of the adopted HA mode on $\dot{V}{\text{O}}_{2\max }$ adaptation (Table 3). This was not anticipated, but was also supported by the finding that neither mechanical intensity nor indices of ambient thermal load during the HA programmes had a moderating effect on $\dot{V}{\text{O}}_{2\max }$ changes (Table 3). Unfortunately, the internal core temperature responses could not be statistically assessed owing to the inconsistent or incomplete reporting of these data but descriptive observation demonstrated no trend in the relationship between $\dot{V}{\text{O}}_{2\max }$ adaptation and core temperature during HA, in any environment (Table 3). Together, these results indicate that the associated increase in mechanical work rates elicited by iso-intensity models (i.e. more intense exercise) or characteristics of the isothermal models, did not influence the level of adaptation observed. These findings are at odds with the theorised necessity of higher-intensity exercise for $\dot{V}{\text{O}}_{2\max }$ adaptation during HA [21]. Indeed, the lack of difference in $\dot{V}{\text{O}}_{2\max }$ adaptation between iso-intensity and iso-thermal designs was not anticipated, as iso-intensity models have been repeatedly shown to augment endurance performance [21, 43, 70], which is not always the case in isothermal studies [27, 41]. It was equally surprising that the lack of moderating effect was consistent for hot $\dot{V}{\text{O}}_{2\max }$ adaptations, since isothermal heat induction is thought to elicit the greatest thermoregulatory effects [14, 71], for the reason that core temperature can be controlled by the investigator during HA [14]. Thus, it is thought that isothermal HA is more likely to enhance the magnitude of daily thermo-effector responses and, in turn, enhances these thermoregulatory defences to a subsequent heat stimulus—as is necessary for the heat acclimated phenotype [12]. Irrespective of this, the current collection of results contradict the seemingly logical inference that isothermal approaches would confer some additional benefit for maximal testing in hot conditions. This could be related to the more recently reported neutral relationship between internal thermal load (time spent > 38.5 °C) and changes in hallmark acclimation responses, such as core temperature or heart rate [29]. Collectively, it is likely that gross, multi-organ systemic outcome measures, such as $\dot{V}{\text{O}}_{2\max }$, require a mixture of thermal and exercise stimuli, which varies between individuals. The recommendation, based on the current evidence, is that the choice of isothermal or iso-intensity will not affect the $\dot{V}{\text{O}}_{2\max }$ outcome in hot or thermoneutral environments.

HA varies in the number of days over which it can be conducted, with short-term heat acclimation (< 7 days) facilitating partial adaptation [72–74], and long-term heat acclimation (often ≥ 7 days) completing this process [75, 76]. This notion was partially supported in the control group meta-analysis of hot $\dot{V}{\text{O}}_{2\max }$ adaption, where the number of HA days significantly moderated the overall effect, such that for every additional day of HA, a 0.29 (*small*) standardised mean increase in $\dot{V}{\text{O}}_{2\max }$ was observed. However, this was not the case in any of the other meta-analyses conducted herein and this particular meta-analysis was based on a total of four articles, which somewhat limits our confidence in the result. Similarly, the ambient temperature of the HA programme also explained significant variance (Table 1) in the outcome of hot $\dot{V}{\text{O}}_{2\max }$ increases in the control group analysis, with hotter temperatures eliciting greater $\dot{V}{\text{O}}_{2\max }$ changes, up to a ceiling value of 40 °C. Thus, notwithstanding the smaller sample of studies, longer and hotter HA programmes appear to confer the greatest effects on hot $\dot{V}{\text{O}}_{2\max }$ when compared to control groups. This could also relate to the specificity of the stimulus and the total heat exposure [64, 65] and agree with the notion that longer adaptation periods might be necessary for full hot adaptation [75, 76]. Interestingly, a recent meta-analysis reported no moderating effect of induction length (HA days) or any other parameter of HA programmes on $\dot{V}{\text{O}}_{2\max }$ adaptation [32] but the number of studies considered for analysis was markedly less and not sub-analysed in the same manner as the current analysis. For example, $\dot{V}{\text{O}}_{2\max }$ assessments in hot and thermoneutral environments were amalgamated for analysis, which will produce mixed results in comparison to the current analysis, since these tasks offer distinctly different challenges.

In the meta-analysis of within-group hot $\dot{V}{\text{O}}_{2\max }$ adaptation, we found that the post-acclimation testing period was significantly related to the outcome. Across the six studies in this analysis, we report that for every additional post-testing day immediately after the final HA intervention, a 0.17 (*small*) standardised change in $\dot{V}{\text{O}}_{2\max }$ can be expected, up to a seven-day limit. In other words, testing or planned performance too close to the final day of HA is not advisable if complete hot $\dot{V}{\text{O}}_{2\max }$ adaptation is to be realised. Extending this up to 7 days appears to be optimal. It should be acknowledged, however, that reporting of this was inconsistent, with many identifying the exact number of days and others stating a time period (i.e. within 7 days). Therefore, there is likely to be some variability in this moderator. Nevertheless, this is an important finding for those utilising heat acclimation to prepare for endurance exercise in hot environments and means that the suggested urgency (see [65]) to acclimate/acclimatise near to competition might be unwarranted if $\dot{V}{\text{O}}_{2\max }$ is considered to be of greatest importance. This is inconsistent with the immediate decays reported in other physiological measures, such as core temperature and heart rate, following HA [12, 73, 77] and is likely to be related to the delayed adaptive responses (absence of immediate decay) to heat acclimation that have been demonstrated in some studies [21, 78, 79]. This phenomenon can be likened to classical dose–response theories of Seyle [80] and explained directly by the severity of the imposed internal thermal load and necessary recovery that subsequently ensues to permit phenotypic adaptation [81]. Given that this was observed in the within-participant hot $\dot{V}{\text{O}}_{2\max }$ analysis, rather than the thermoneutral equivalents, it is possible that the specificity of the hot environment during post-testing underpins this theory. However, experimental work has recently demonstrated a similar pattern of adaptation following heat acclimation when testing in thermoneutral environments [21]. Therefore, further work is needed to understand this phenomenon and its physiological determinants.

As we anticipated, there were numerous inconsistencies between studies, which limit some of the conclusions of the current meta-analyses and should be considered when using these results to inform HA programme design. For example, core body temperature in response to HA sessions was often reported as either a mean or a final temperature reached. Whilst there is typically a relationship between these variables, it would be helpful for readers if more complete reporting of the mean, standard deviation and final core body temperatures is provided. In addition, the measurement of core body temperature (rectal, oesophageal, tympanic, ingestible pill) often varies between studies and could alter the magnitude of the response. Whilst we used SMD to control for differences in measurement type in the current meta-analyses, readers should be aware of this when evaluating the raw data we presented herein from previous studies. Finally, the number of days between HA completion and post-testing of $\dot{V}{\text{O}}_{2\max }$ should be reported more consistently among studies, perhaps through submission of raw data to support summary findings, since this appears to affect the $\dot{V}{\text{O}}_{2\max }$ measurement. More exact reporting of this would help to understand the consistency of this conclusion and improve the design of HA programmes, if $\dot{V}{\text{O}}_{2\max }$ improvement is assumed to be a desirable outcome.

## Conclusion

The collective conclusions drawn from the current meta-analyses are that HA can enhance $\dot{V}{\text{O}}_{2\max }$ adaptation in thermoneutral or hot environments by at least a *small* and up to a *moderate-large* amount, with the descriptively larger improvements occurring in the heat. The positive effects of HA on $\dot{V}{\text{O}}_{2\max }$ were maintained with or without the inclusion of a control group, suggesting its capacity to augment the effect of endurance training. The type of programme adopted (isothermal or iso-intensity) did not appear to affect the training adaptation but the ambient heat and number of induction days do explain the change in hot $\dot{V}{\text{O}}_{2\max }$ adaptation, which could support the necessity of higher thermal volumes (exposures) and similarity of the training to the testing environment to maximise adaptation. The number of post-testing days also appears to play a role in hot $\dot{V}{\text{O}}_{2\max }$ adaptation and further work is required to explain the underlying physiology of this delayed response.

## Declarations

### Availability of data and material

Available on request.
